# Inhibition of inflammasome activation by a clinical strain of *Klebsiella pneumoniae* impairs efferocytosis and leads to bacterial dissemination

**DOI:** 10.1038/s41419-018-1214-5

**Published:** 2018-12-05

**Authors:** Ana Campos Codo, Amanda Correia Saraiva, Leonardo Lima dos Santos, Marina Francisco Visconde, Ana Cristina Gales, Dario Simões Zamboni, Alexandra Ivo Medeiros

**Affiliations:** 1 Department of Biological Sciences, School of Pharmaceutical Sciences, São Paulo State University, Araraquara, São Paulo Brazil; 2 Department of Cell and Molecular Biology and Pathogenic Bioagents, School of Medicine, São Paulo University, Ribeirão Preto, São Paulo Brazil; 3 Division of Infectious Diseases, Department of Internal Medicine, Escola Paulista de Medicina, UNIFESP, São Paulo, São Paulo Brazil

## Abstract

*Klebsiella pneumoniae* is a Gram-negative bacterium responsible for severe cases of nosocomial pneumonia. During the infectious process, both neutrophils and monocytes migrate to the site of infection, where they carry out their effector functions and can be affected by different patterns of cell death. Our data show that clinical strains of *K. pneumoniae* have dissimilar mechanisms for surviving within macrophages; these mechanisms include modulation of microbicidal mediators and cell death. The A28006 strain induced high IL-1β production and pyroptotic cell death in macrophages; by contrast, the A54970 strain induced high IL-10 production and low IL-1β production by macrophages. Pyroptotic cell death induced by the A28006 strain leads to a significant increase in bacterial sensitivity to hydrogen peroxide, and efferocytosis of the pyroptotic cells results in efficient bacterial clearance both in vitro and in vivo. In addition, the A54970 strain was able to inhibit inflammasome activation and pyroptotic cell death by inducing IL-10 production. Here, for the first time, we present a *K. pneumoniae* strain able to inhibit inflammasome activation, leading to bacterial survival and dissemination in the host. The understanding of possible escape mechanisms is essential in the search for alternative treatments against multidrug-resistant bacteria.

## Introduction

Macrophages are known to play an important role in host defense against different pathogens by producing reactive oxygen and nitrogen species as well as inflammatory cytokines^1,2^. Indeed, macrophage depletion compromises efficient clearance of pathogens^3,4^. *Klebsiella pneumoniae* is a Gram-negative capsulated bacterium responsible for infections at several sites in the host organism, especially the pulmonary and urinary tracts, being considered a major pathogen for nosocomial pneumonia^5^ and a main source of sepsis^6^. In immunocompromised patients, *K. pneumoniae* infections can be particularly devastating, with high mortality rates^7^. In addition to having several mechanisms to evade the activity of antibiotics such as carbapenems, as well as antimicrobial compounds, *K. pneumoniae* is also able to escape from the phagolysosome to the cytosol. In the cytosol, the bacteria can interact with cytosolic pattern recognition receptors (PRRs), especially Nod-like receptors (NLRs). NLRs are known to detect bacterial products introduced into the host cytosol, such as bacterial wall peptidoglycans^8^, as well as endogenous danger signals^9,10^. Engagement of these receptors may trigger inflammasome activation, leading to caspase-1 activation and interleukin (IL)-1β production. Non-canonical inflammasome activation through caspase-11 recognition of Gram-negative bacterial lipopolysaccharide had its importance increasingly recognized^11^, and a recent study showed the role of caspase-11 in *K. pneumoniae* infections^12^. In addition, it is well described that *K. pneumoniae* is able to activate NLRP3 and NLRC4 inflammasomes^13–15^.

During pathogen evolution, several molecular mechanisms were acquired, allowing them to escape inflammasome activation. For instance, an elegant study showed that flagellin-deficient *Legionella pneumophila* mutants avoids caspase-1 activation, thereby avoiding inflammasome formation, culminating in the survival of the bacteria against the host immune response^16^. In addition to avoiding caspase-1 activation, some pathogens can also inhibit inflammasome activation. Cunha et al. demonstrate that *Coxiella burnetii* can inhibit the caspase-11-mediated non-canonical activation of the NLRP3 inflammasome^17^.

Inflammasome activation requires two signals. The first signal is produced by PRRs and leads to activation of transcription factors such as nuclear factor-κB and activator protein-1. These factors will then transcribe NLRs, pro-caspase-1 and pro-IL-1β, as well as several proinflammatory cytokines, such as tumor necrosis factor (TNF)-α and IL-12^18^. However, a second stimulus is required for NLR activation, which results in the cleavage of caspase-1 together with protein recruitment, forming the inflammasome and secreting active IL-1β^19^. IL-1β has been described to play an important role in host defense, enhancing the phagocytic capacity of macrophages and the production of chemokines such as IL-8, in addition to being involved in neutrophil and monocyte infiltration to the site of infection^14,20,21^. Aside from IL-1β production, activation of caspase-1 also triggers a form of cell death called pyroptosis^22^. In contrast to apoptosis, pyroptotic cell death induces the release of proinflammatory mediators due to the formation of cell membrane pores and the release of soluble cytosolic contents^23^. Different pathogens are able to induce pyroptotic cell death, which contributes to the host defense mechanism against infection^24–27^. However, some bacteria are able to avoid pyroptosis as a strategy to evade the host defenses. During *Salmonella* Typhimurium infection, pyroptotic macrophages that have engulfed the bacteria release their intracellular components, contributing to an inflammatory response and recruitment of new phagocytes to engulf the damaged bacteria trapped in cell corpses^28^. The engulfment of dead cells, termed efferocytosis, is essential to restore tissue homeostasis during apoptotic tissue renewal or injury response^29^. Moreover, efferocytosis of pyroptotic cells seems to play an essential role in host defense against *S.* Typhimurium by increasing bacterial damage due to contact with microbicidal factors^30^.

The current study focused on two genetically similar *K. pneumoniae* strains isolated from patients’ bloodstream. Clinical isolates of *K. pneumoniae* are frequently resistant to multiple antibiotics^31^, and both strains used in this study are multidrug resistant. This stresses the importance of understanding the innate immune defense against infections caused by *K. pneumoniae* carbapenemase (KPC)-producing bacteria, which could help the development of new therapeutic strategies.

*K. pneumoniae* clinical strains have several mechanisms to survive within macrophages, including modulation of microbicidal mediators and cell death. Here we demonstrate for the first time that efferocytosis of pyroptotic cells has a protective effect on host defense toward *K. pneumoniae* clinical strains, since its inhibition allows in vivo bacterial dissemination.

## Results

### Two clinical KPC-2-producing *K. pneumoniae* strains have opposite behavior in vitro

**Fig. 1:**
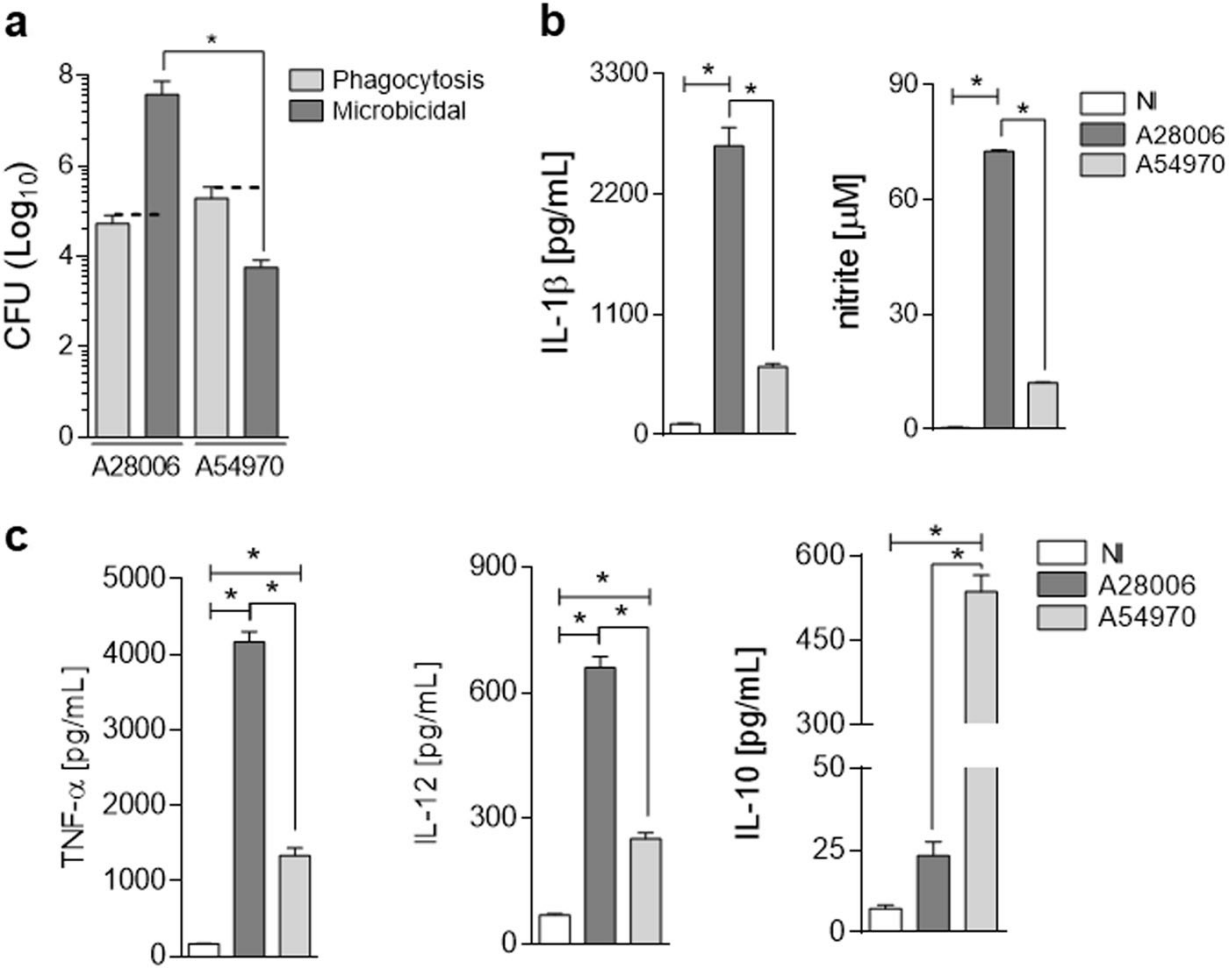
*K. pneumoniae* strains are not eliminated by macrophages and induce different profiles of mediator production. BMDMs were differentiated from C57BL/6 mice and incubated with the A28006 or the A54970 strains (MOI 1:5) for 90 min. Cells were washed twice with a cocktail of antibiotics in PBS and the last wash with PBS. **a** For the phagocytosis assay, cells were lysed with saponin and CFU was recovered. For the microbicidal assay, cells were incubated for 4 h more before cell lysis and CFU recovery. **b**, **c** BMDM were infected with the A28006 or the A54970 strain (MOI 1:5) and after 18 h the supernatant was collected. **b** IL-1β was quantified by ELISA and NO was indirectly quantified by Griess reaction. **c** TNF-α, IL-12, and IL-10 were quantified by ELISA. **a**–**c** Data represent mean ± SEM of at least three independent experiments performed in triplicate. Statistical analysis: one-way ANOVA and Tukey post hoc tests. *P < 0.05

Several pathogens are able to evade the phagocytic and macrophage microbicidal mechanisms imposed by diverse phagocytes through the possession of a polysaccharide capsule and molecular modifications in lipopolysaccharide (LPS)^32^. Considering that *K. pneumoniae* is a capsulated Gram-negative bacterium, we first evaluated the phagocytic capacity of macrophages toward two different KPC-2-producing clinical strains of *K. pneumoniae*, A28006 and A54970. Bone marrow-derived macrophages (BMDMs) were incubated with A28006 or A54970, and phagocytic capacity was evaluated by colony-forming unit (CFU) recovery and confirmed by flow cytometry. The phagocytic capacity of BMDMs was similar in the presence of both strains of *K. pneumoniae* (Fig. 1a and S1a). Regarding microbicidal capacity, BMDMs were not able to eliminate either strain of *K. pneumoniae*. However, the A54970 strain seems to be more susceptible than the A28006 strain to microbicidal activity; the latter strain was able to replicate inside the phagocytes (Fig. 1a).

Since we did not observe bacterial elimination with either strain, we evaluated the pattern of mediators produced during in vitro infection. A28006 infection induces increased amounts of IL-1β production, while infection with A54970 strain induces no production of this mediator (Fig. 1b). In addition, we observed that BMDMs infected with the A28006 strain produce larger amounts of nitric oxide (NO) (Fig. 1c), TNF-α, and IL-12 (p70) than the A54970 strain does. Furthermore, production of IL-10 by macrophages is markedly higher in A54970 infection than in A28006 infection (Fig. 1c).

Since A54970 strain failed to trigger IL-1β production, induced high levels of IL-10 and seems to be more susceptible than A28006 strain to microbicidal activity, we investigated the polarization of macrophages into M1 and M2 phenotype post bacterial infection. Despite the IL-10 production, macrophages infected with both strains have similar M2 marker expression, such as *Arg1*, *Mrc1* (CD206), and *Fizz-1*, and as expected, A28006 strain induced higher expression of *Nos2* and *Il-6* (Figure S2).

These data indicate that the A28006 strain promotes inflammasome activation that triggers high IL-1β production, while the A54970 strain was unable to do so.

### *K. pneumoniae* strains induce different patterns of cell death

**Fig. 2:**
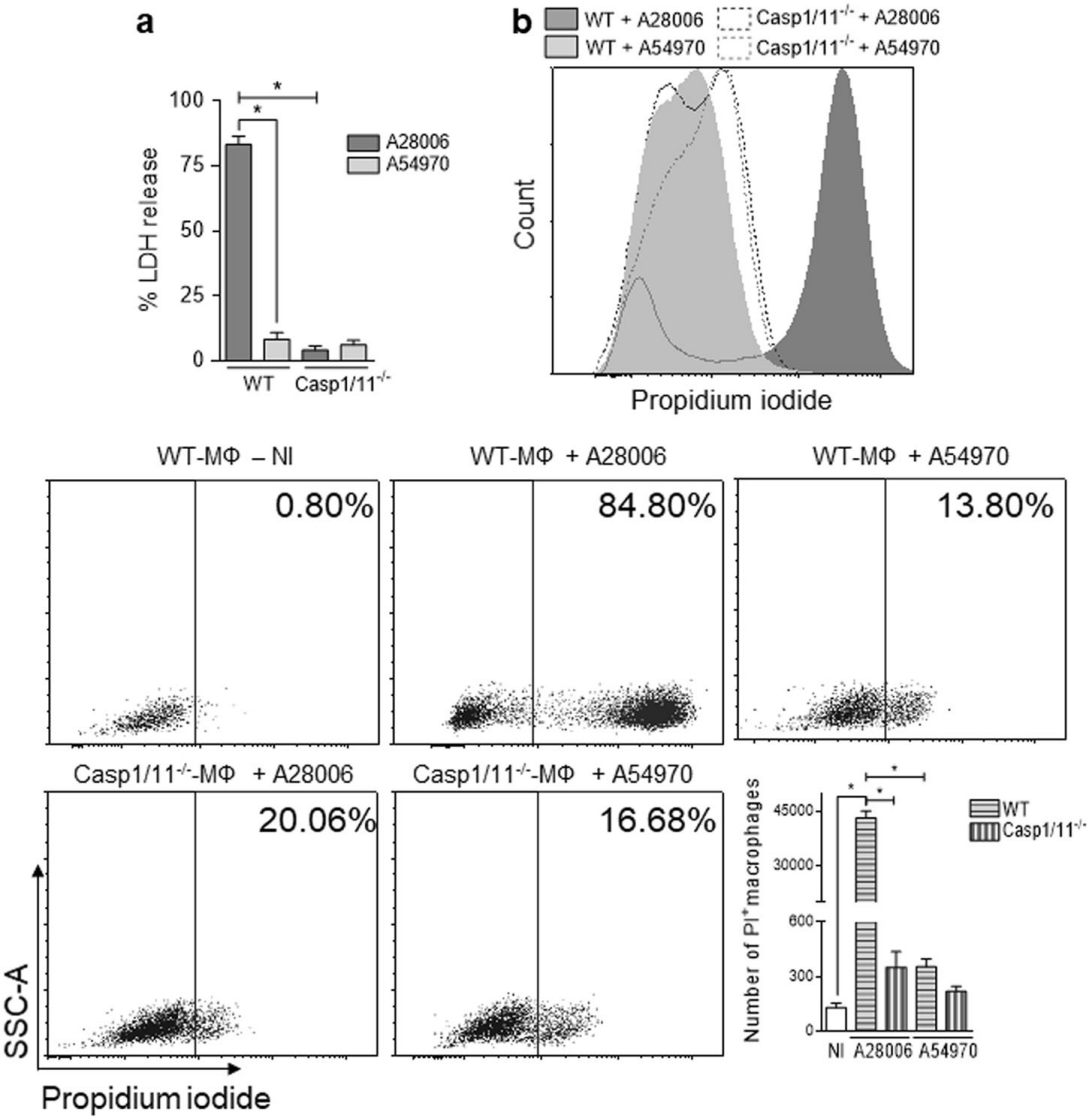
A28006 and A54970 strains induce different cell death patterns in macrophages. BMDMs were differentiated from WT and Casp1/11^−/−^ C57BL/6 mice and incubated with A28006 or A54970 strains (MOI 1:5) for 90 min. Cells were washed twice with a cocktail of antibiotics in PBS and one last wash with PBS. Cells were incubated for 4 h more and membrane permeability was evaluated by **a** LDH release in the supernatant and **b** by PI incorporation. The number of PI^+^ macrophages was determined by flow cytometry and is shown by a representative dot plot of three independent experiments. Data represent the mean ± SEM of three independent experiments performed in triplicate. Statistical analysis: one-way ANOVA and Tukey post hoc tests. *P < 0.05

The production of IL-1β and IL-18 requires inflammasome activation, and pyroptosis has been suggested as a requirement for the release of those two ILs^33,34^. We sought to investigate whether the difference in IL-1β production induced by these two strains of *K. pneumoniae* could be related to different patterns of cell death. As expected, A28006 infection in BMDMs led to higher amounts of lactate dehydrogenase (LDH) release (Fig. 2a) and propidium iodide (PI) incorporation (Fig. 2b and Figure S3) than A54970 infection did.

We next examined whether this loss of membrane integrity was due to pyroptosis by using BMDMs from caspase-1 and caspase-11 knockout mice to impair inflammasome activation. The absence of caspase-1/11 drastically inhibited LDH release (Fig. 2a) and PI incorporation (Fig. 2b) from BMDMs infected with A28006. However, infection with A54970 had no effect on the Casp1/11^−/−^ BMDMs. Moreover, the absence of Casp1/11 compromised neither phagocytosis nor microbicidal abilities (Figure S1b, c). Apart from IL-1β, the absence of these proteases had no effects in the production of other mediators during infection with either strain (data not shown). These results demonstrate that different strains of *K. pneumoniae* induce distinct patterns of cell death. While the A28006 strain promotes pyroptosis and robust levels of IL-1β in macrophages, the A54970 strain has a modest effect on these events.

### Cell death by pyroptosis increases the bacteria susceptibility to hydrogen peroxide

**Fig. 3:**
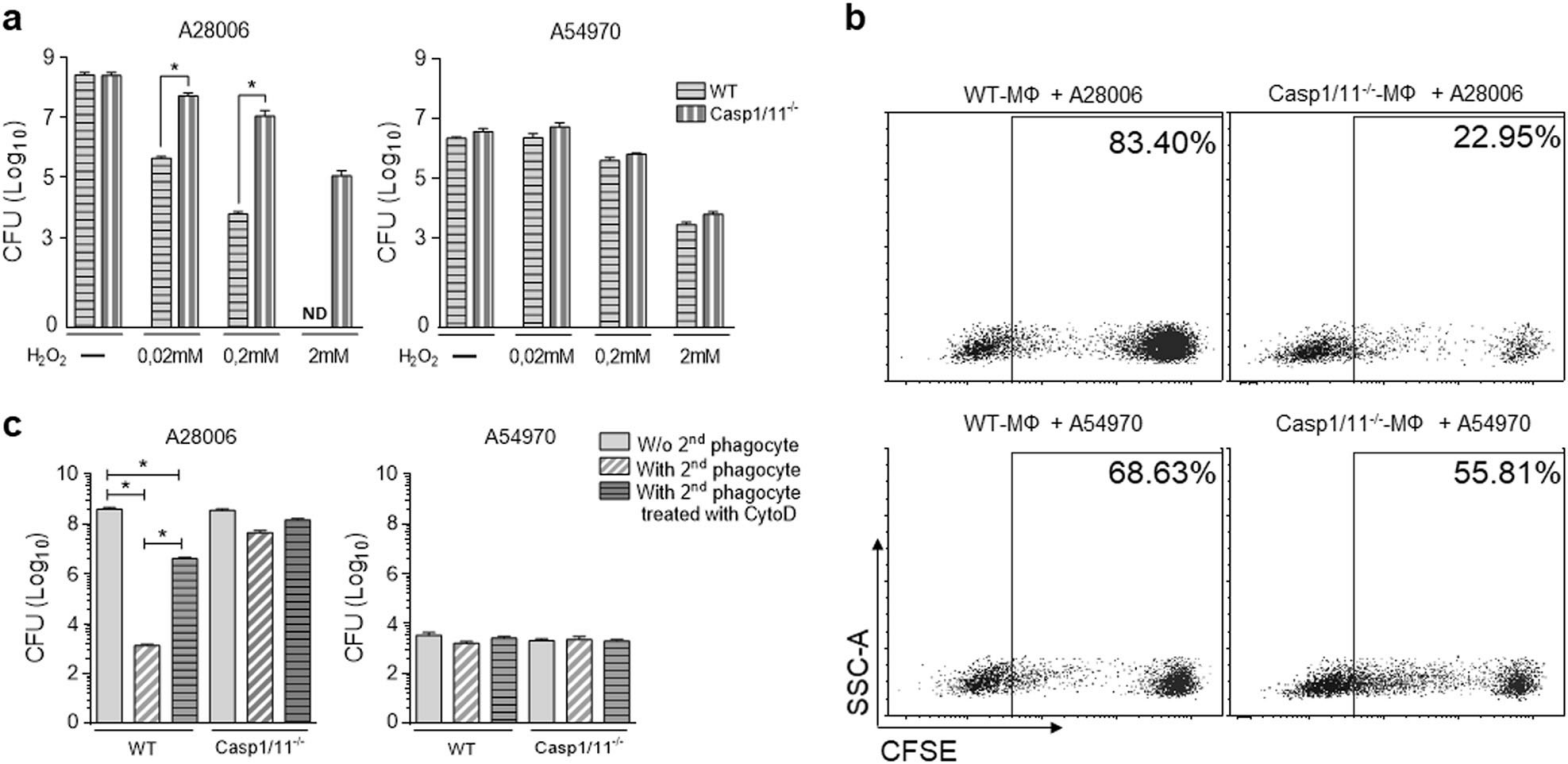
Pyroptosis leads to elevated microbicidal susceptibility, favoring clearance by efferocytosis. BMDMs were differentiated from WT and Casp1/11^−/−^ mice and incubated with the A28006 or A54970 strains (MOI 1:5) for 90 min. Cells were washed twice with a cocktail of antibiotics in PBS, one last wash with PBS, and incubated for 4 h more. **a** For CFU recovery, cells were lysed with saponin and incubated for 2 h in the absence or presence of different concentrations of hydrogen peroxide. **b** CFSE-labeled macrophages were infected with the A28006 or A54970 strains (MOI 1:5). After 4 h of incubation, new unlabeled WT BMDMs were co-cultured with infected cells for 2 h in a ratio 1:3 (new BMDMs:infected cells). Non-phagocytosed cells were quenched and washed with PBS. The percentage of CFSE^+^ macrophages was determined by flow cytometry and is shown by a representative dot plot of three independent experiments. **c** New BMDMs were incubated in the presence or absence of cytochalasin D for 30 min and then co-cultured with infected cells for 2 h in a ratio 1:3. Bacterial clearance through efferocytosis was assessed by CFU recovery. **a**, **c** Data represent mean ± SEM of three independent experiments performed in triplicate. Statistical analysis: one-way ANOVA and Tukey post hoc tests. ND not detected. *P < 0.05

Some studies have reported that pyroptosis traps bacteria inside pore-induced intracellular trap (PITs), making the bacteria more susceptible to other microbicidal stressors^30^. Since we observed that the A54970 strain was unable to induce pyroptosis, we sought to investigate whether this pattern of cell death might be facilitating the resolution of infection. To assess whether A54970 would be more resistant than A28006 to bactericidal agents, we cultivated bacteria from cell lysates of infected BMDMs in different concentrations of H_2_O_2_. A28006 strain had higher susceptibility to hydrogen peroxide stress than A54970 did, and this effect was reversed when inflammasome activation was impaired in Casp1/11^−/−^ macrophages (Fig. 3a). By contrast, the absence of caspase-1/11 had no effect on the susceptibility of A54970 strain to H_2_O_2._ This suggests that inflammasome activation and pyroptosis triggered by the A28006 strain leads to hydrogen peroxide susceptibility, in contrast to the A54970 strain, which does not trigger pyroptosis.

### Efferocytosis of infected pyroptotic cells improves bacterial clearance

Similar to apoptotic bodies, pyroptotic cells expose “find-me” and “eat-me” signals that promote efferocytosis by phagocytes^30^. Since efferocytosis of infected cells plays a critical role in host defense against bacterial infection, we hypothesized that the engulfment of bacteria containing PITs would lead to bacterial clearance. To assess whether efferocytosis could improve the resolution of infection, we co-cultured infected BMDMs with freshly collected BMDMs, after which we evaluated efferocytosis and bacterial clearance. Pyroptotic cell death induced by the A28006 strain led to an effective engulfment by macrophages in a caspase-1/11-dependent manner (Fig. 3b) and promoted efficient bacterial clearance. However, efferocytosis of A54970-infected macrophages had no effect on bacterial count (Fig. 3c).

To confirm that efficient bacterial clearance was due to efferocytosis of A54970-infected macrophages by BMDMs, we used cytochalasin D (Cyto D) to block the phagocytosis of infected cells by other phagocytes. Cyto D was able to inhibit nearly 80% of efferocytosis (Figure S4a), and the inhibition of efferocytosis of A28006-infected cells inhibited bacterial clearance (Fig. 3c). Despite the difference in efferocytosis rates, there were no differences in hydrogen peroxide production by efferocytic BMDMs, showing that bacterial elimination occurred through the function of efferocytosis itself (Figure S4b).

Through CFU recovery, we observed that the A28006 strain was nearly eliminated by efferocytosis in wild-type (WT) BMDMs, while blockage of pyroptosis or efferocytosis allowed bacteria proliferation. In addition, the A54970 strain, which is unable to induce pyroptosis, was able to proliferate in BMDMs. These results suggest that efferocytosis could be the major mechanism of bacterial elimination.

### Efferocytosis of infected pyroptotic cells improves resolution of in vivo infection

**Fig. 4:**
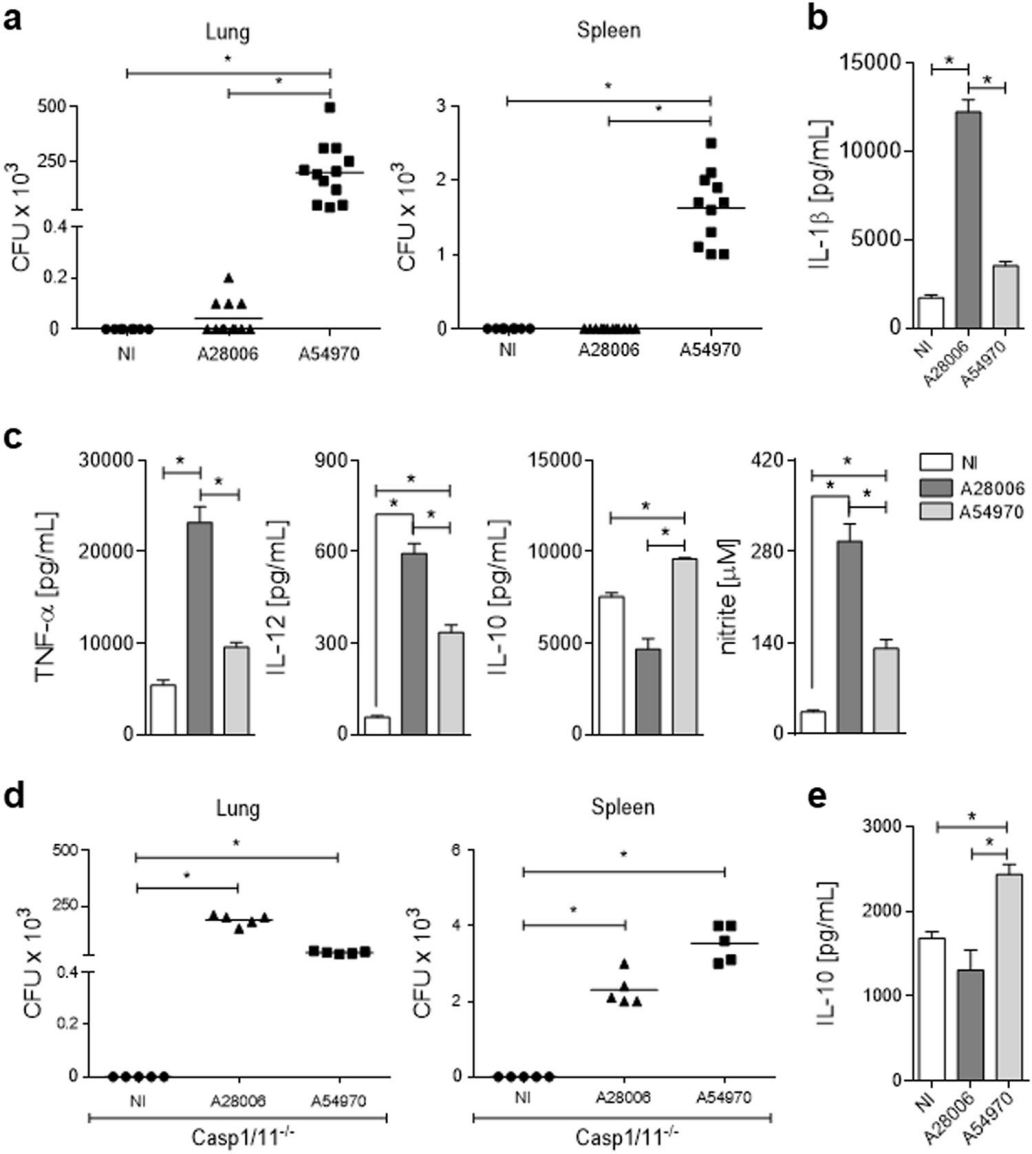
Pyroptosis improves the resolution of in vivo infection. **a**–**c** WT C57BL/6 mice were nasally instilled with 1 × 10^7^ CFU. After 48 h of inoculation, the lungs and spleen were removed and homogenized for CFU recovery and cytokine determination. **a** Bacterial load in the lung and spleen of infected mice. **b**, **c** Cytokines determined in lung homogenate. **b** IL-1β, **c** TNF-α, IL-12, and IL-10 were quantified by ELISA. Nitrite was quantified by Griess reaction. **a**–**c** Data represent the mean ± SEM of two of three independent experiments (*n* = 6 animal/group). Statistical analysis: one-way ANOVA and Tukey post hoc tests. **d**, **e** Casp1/11^−/−^ mice were nasally instilled with 1 × 10^7^ CFU. After 48 h of inoculation, the lungs and spleen were removed and homogenized with a tissue homogenizer in the presence of protease inhibitor. **d** Bacterial load in the lung and spleen of infected mice. **e** Supernatant of lung homogenate was collected and IL-10 was quantified by ELISA. **d**, **e** Data represent the mean ± SEM of one independent experiment (*n* = 5 animal/group). Statistical analysis: one-way ANOVA and Tukey post hoc tests. *P < 0.05

Next, we hypothesized that the A54970 clinical isolate of *K. pneumoniae* could disseminate in vivo and cause illness in the host. Therefore, we evaluated bacterial dissemination through CFU recovery in the lungs and spleen of C57BL/6 infected mice. Interestingly, the A54970 strain demonstrated higher survival in the lungs, as well as higher in vivo dissemination capacity, than the A28006 strain (Fig. 4a). In addition, A54970 infection caused a 40% loss of animal weight during the infection, representing more severe illness (Figure S5).

Consistent with in vitro observation, when mice were infected with the A28006 strain, we observed higher levels of IL-1β than we observed in pulmonary A54970 infection (Fig. 4b). Additionally, A28006 strain infection led to increased amounts of TNF-α and IL-12, as well as microbicidal NO, compared to the amounts induced by A54970 infection. Surprisingly, production of IL-10 in the lungs of A54970 infected mice was notably two-fold higher than A28006 infection. (Fig. 4c).

Thus, to confirm that pyroptosis leads to in vivo bacterial clearance through efferocytosis, Casp1/11^−/−^ mice were infected with both strains and bacterial load was evaluated in the lung and spleen. The absence of Casp1/11 impairs pyroptosis cell death and the A28006 strain was able to proliferate in the lung and disseminate to spleen, as observed in the A54970 strain (Fig. 4d). As expected, IL-1β production was inhibited in knockout animals (data not shown) and had no effect on the IL-10 production (Fig. 4e). Therefore, we confirmed that pyroptosis-escaping mechanisms results in bacterial dissemination.

### The A54970 strain does not induce IL-1β production or pyroptosis in response to LPS stimulus

**Fig. 5:**
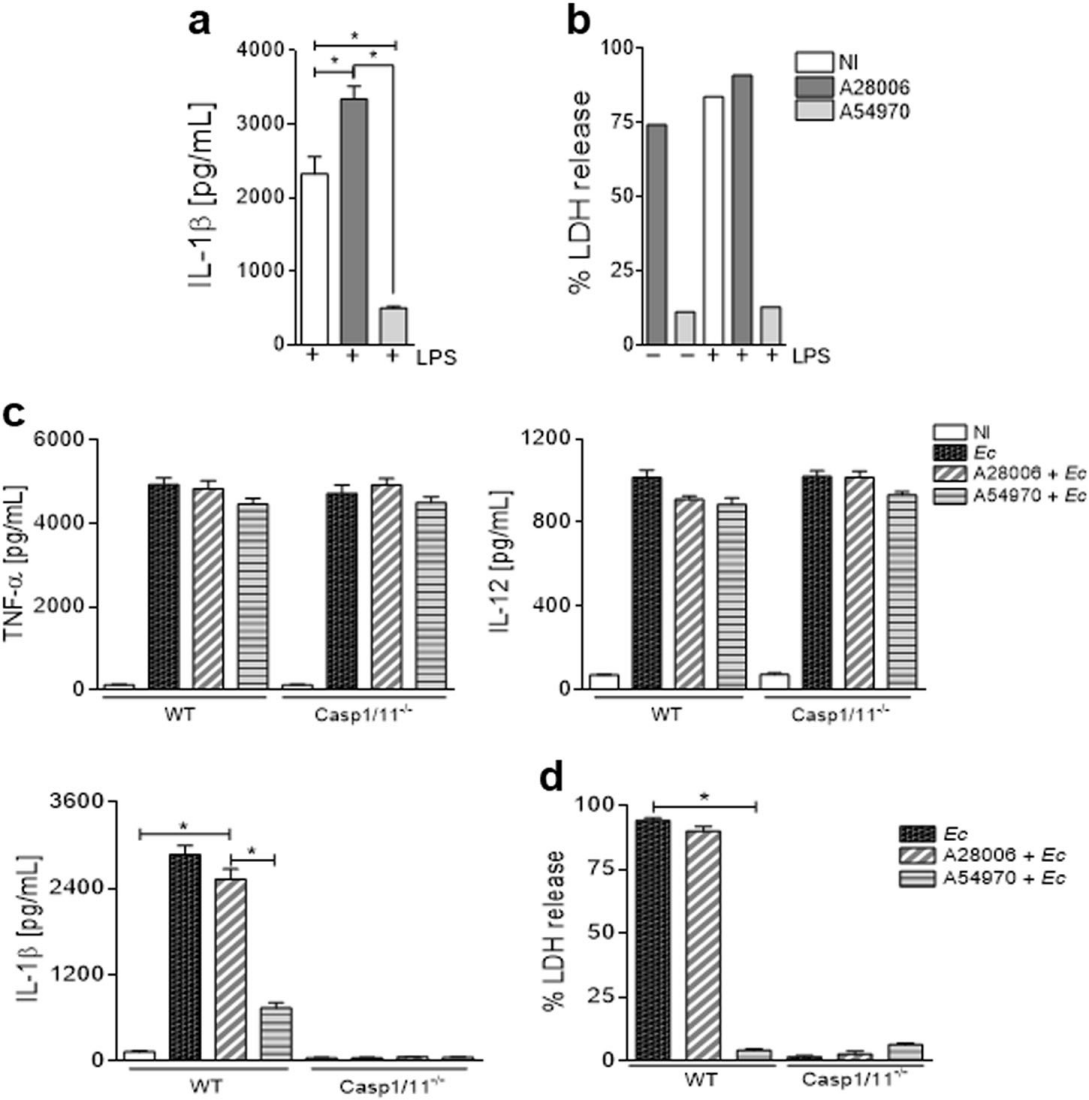
The A54970 strain inhibits inflammasome activation and pyroptosis. **a**, **b** BMDMs differentiated from WT were incubated or not with 100 ng.mL^−1^ of LPS for 3 h and then infected with the A28006 or the A54970 strain (MOI 1:5) for 90 min. Cells were washed twice with a cocktail of antibiotics in PBS, one last wash with PBS. After 18 h, the supernatant was collected. **a** IL-1β levels were measured by ELISA and **b** LDH release was measured in the supernatant. **c**, **d** BMDMs from WT and Casp1/11^−/−^ mice were incubated with the A28006 or the A54970 strains (MOI 1:5) for 90 min. Cells were incubated for 24 h more. After then, cells were infected with *E. coli* (MOI 1:20) for 4 h and the supernatant was collected for **c** TNF-α, IL-12, and IL-1β levels determination by ELISA. **d** Cell lysis was evaluated by LDH release in the supernatant. **a** Data represent the mean ± SEM of one independent experiment performed in triplicate. **b** Data represent one independent experiment. **c**, **d** Data represent the mean ± SEM of three independent experiments performed in triplicate. Statistical analysis: one-way ANOVA and Tukey post hoc tests. *P < 0.05

To understand the modest effect of A54970 infection, we evaluated whether the A54970 strain was not able to induce an efficient priming signal compared to A28006 strain. To prove this hypothesis, macrophages were incubated with LPS for 3 h prior to bacterial infection and IL-1β production and LDH release were evaluated. Even then, in the presence of LPS stimulus, macrophages infected with A54970 strain were not able to produce IL-1β (Fig. 5a). Similarly, A54970 strain fails in inducing pyroptosis independently of LPS stimulus (Fig. 5b). These data showed that A54970 strain inhibits the signaling through Toll-like receptor that could impair IL-1β production and LDH release.

### The A54970 strain inhibits inflammasome activation and pore formation in response to *Escherichia coli*

Since the A54970 strain was unable to induce IL-1β production by macrophages, we sought to determine whether this strain is able to inhibit the priming of the cells through inhibition of NLR transcription or through inhibition of inflammasome activation itself. Therefore, we evaluated whether TNF-α and IL-12 or IL-1β production were inhibited by the A54970 strain. BMDMs were infected with both strains and then incubated with *E. coli*, which is known to activate the inflammasome and induce pyroptosis^17^. BMDMs co-infected with A54970 and *E. coli* were able to produce TNF-α and IL-12 (Fig. 5c), demonstrating that theses macrophages were efficiently primed. However, IL-1β production was inhibited regardless of *E. coli* stimuli (Fig. 5c). Moreover, in the presence of *E. coli*, the A54970 strain impaired LDH release from BMDMs, suggesting pyroptosis inhibition (Fig. 5d). These results suggest that A54970 inhibits inflammasome activation and cell death by pyroptosis, thereby preventing bacterial clearance through efferocytosis.

### A54970 inhibits inflammasome activation through IL-10 production

**Fig. 6:**
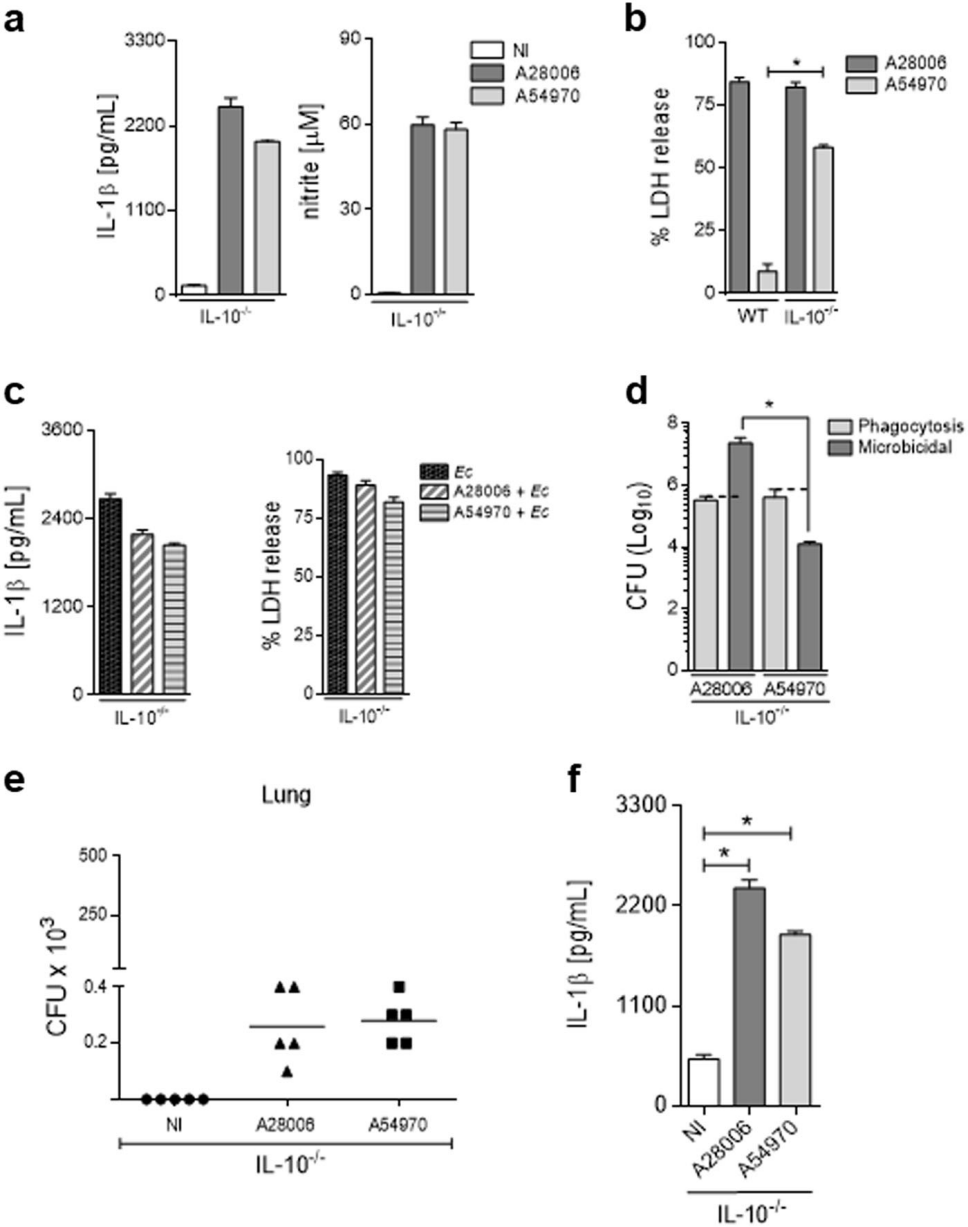
The A54970 strain inhibits inflammasome activation through induction of IL-10. BMDMs were differentiated from WT and IL-10^−/−^ mice and incubated with the A28006 or the A54970 strain (MOI 1:5) for 90 min. Cells were washed twice with a cocktail of antibiotics in PBS, one last wash with PBS. **a** Cells were incubated for 18 h, and the supernatant was collected, IL-1β was quantified by ELISA and Nitrite was quantified by Griess reaction. **b** Cell were incubated for 4 h and LDH release was measured in the supernatant. **c** Cells were incubated for 24 h more and infected with *E. coli* (MOI 20) for 4 h. The supernatant was collected for IL-1β quantification by ELISA and LDH release. **d** For the phagocytosis assay, cells were lysed with saponin and CFU was recovered. For the microbicidal assay, cells were incubated for 4 h more, then lysed with saponin and CFU was recovered. **a**–**d** Data represent the mean ± SEM of three independent experiments performed in triplicate. Statistical analysis: one-way ANOVA and Tukey post hoc tests. **e**, **f** IL-10^−/−^ mice were nasally instilled with 1 × 10^7^ CFU. After 48 h of instillation, the lungs and spleen were removed and **e** bacterial load was determined in tissue homogenates. **f** Supernatant of lung homogenate was collected and IL-1β was quantified by ELISA. **e**, **f** Data represent the mean ± SEM of one independent experiment (*n* = 5 animal/group). Statistical analysis: one-way ANOVA and Tukey post hoc tests. *P < 0.05

It has been recently described that IL-10 is able to induce mitophagy and dysregulate NLRP3 inflammasome activation^35^. We speculated that A54970-mediated inhibition of inflammasome activation might be due to the high levels of IL-10 produced during the in vitro and in vivo infection with this strain. BMDMs from IL-10 knockout mice were infected with A54970 strain, and inflammasome activation was assessed by IL-1β production and LDH release. In the absence of IL-10, macrophages were able to produce IL-1β and NO in response to infection with A54970 strain, in the same levels as induced by BMDMs infected with A28006 (Fig. 6a). Corroborating these results, the A54970 strain induced pyroptosis in IL-10^−/−^ BMDMs, resulting in high LDH release (Fig. 6b). As expected, the A54970 infection of BMDMs from IL-10^−/−^ mice was unable to inhibit *E. coli* response (Fig. 6c); furthermore, their phagocytic and microbicidal abilities were not altered in IL-10^−/−^ cells (Fig. 6d).

Next, to confirm in vivo that IL-10 plays a crucial role to inhibit inflammasome activation and bacterial clearance, IL-10 knockout mice were infected with the A54970 strain and survival and bacterial load were evaluated. Interestingly, the absence of IL-10 during the infection with A54970 strain leads to similar bacterial load in the lungs (Fig. 6e) and no bacterial load in the spleen (data not shown). Moreover, IL-10^−/−^ mice infected with A54970 failed to inhibit inflammasome activation and recover the capacity to produce IL-1β in the lung when compared to non-infected mice (Fig. 6f).

These data further confirm that this *K. pneumoniae* clinical strain is able to inhibit inflammasome activation and its functions through higher induction of IL-10.

### IL-10 inhibits pyroptosis in vitro

**Fig. 7:**
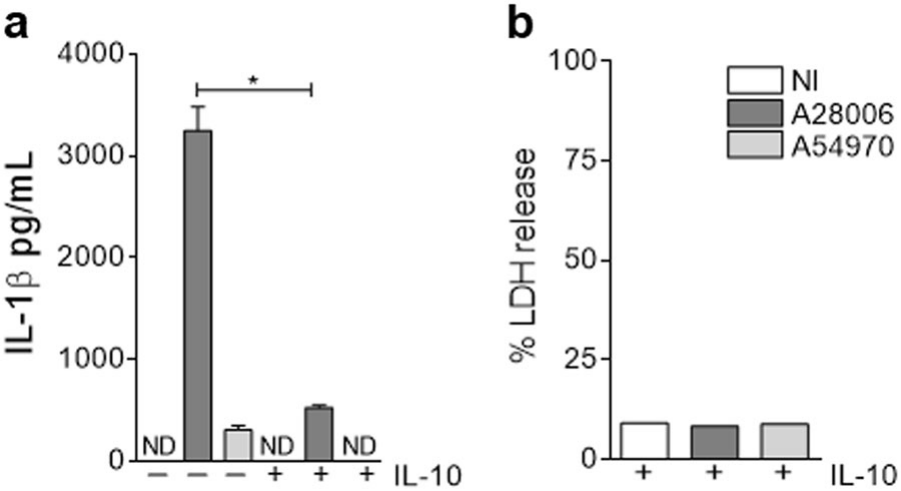
IL-10 inhibits pyroptosis in vitro. **a**, **b** WT BMDMs were incubated in the presence or absence of IL-10 for 24 h, then infected with the A28006 or the A54970 strain (MOI 1:5) for 90 min. Cells were washed twice with a cocktail of antibiotics in PBS, one last wash with PBS, and incubated for 18 h more and the supernatant was collected. **a** Levels of IL-1β were determined by ELISA and **b** LDH release was measured in the supernatant. **a** Data represent the mean ± SEM of one independent experiment performed in triplicate. **b** Data represent values of one independent experiment. Statistical analysis: one-way ANOVA and Tukey post hoc tests. ND not detected. *P < 0.05

To confirm that IL-10 is the mechanism by which pyroptosis is inhibited, BMDMs were pretreated with IL-10 during 24 h prior to *K. pneumoniae* clinical strain infection. We verified that IL-10 was able to inhibit inflammasome activation by A28006 infection, resulting in low levels of IL-1β as well as LDH production (Fig. 7a, b). These results demonstrate that IL-10 is able to inhibit pyroptosis despite the infection stimuli with *K. pneumoniae* clinical strain.

**Fig. 8:**
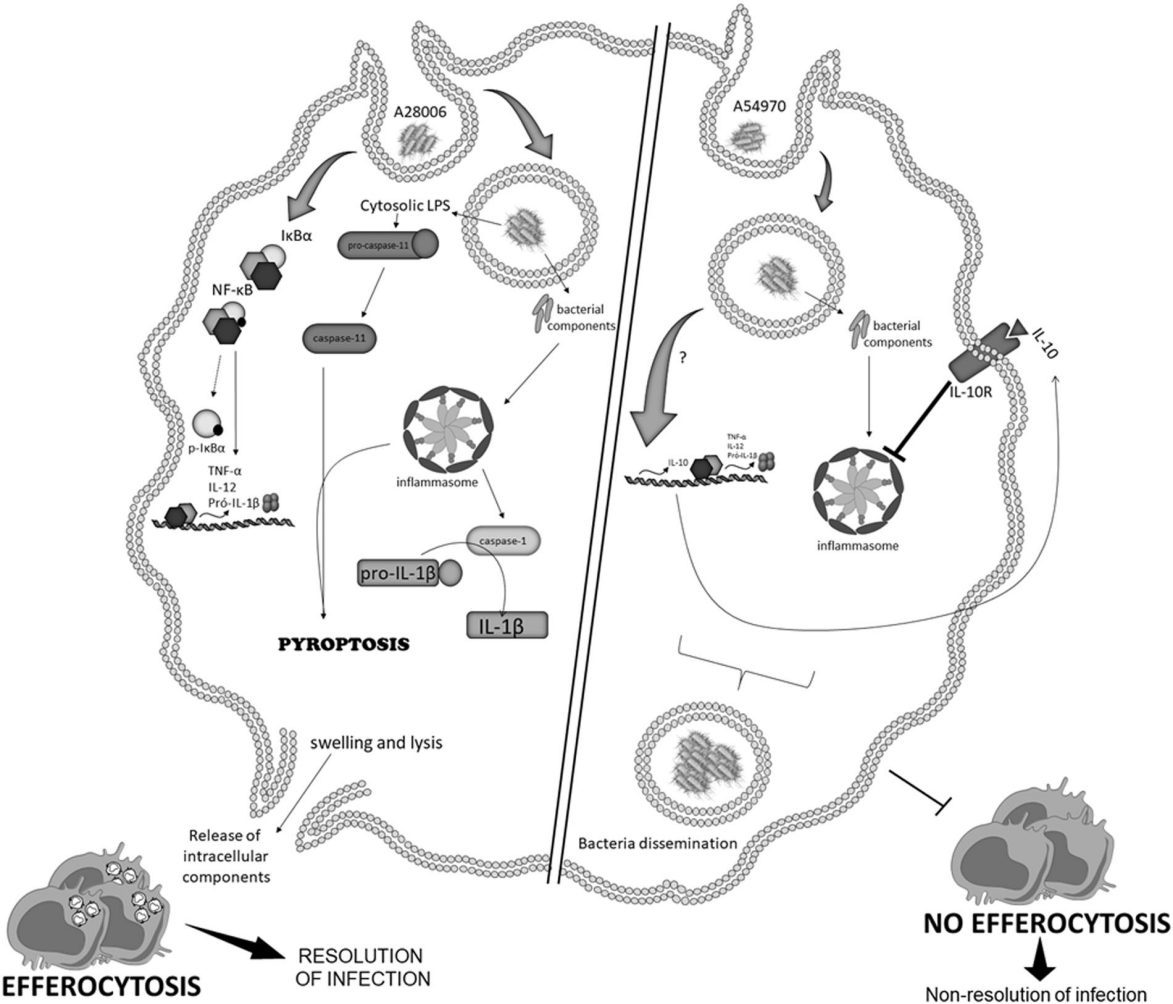
*Klebsiella pneumoniae*-mediated induction of protective pyroptosis via inflammasome activation. The A28006 strain activates inflammasome through bacterial components and caspase-11, triggering inflammasome formation, which allows caspase-1 activation. Therefore, there is the cleavage of pro-IL-1β in mature IL-1β. In addition, there is induction of cell death by pyroptosis, culminating in pore formation and release of intracellular soluble components, such as LDH. IL-1β together with other mediators are capable to recruit new cells to the infection site, promoting efferocytosis of pyroptotic-infected cells and bacterial clearance. Nevertheless, IL-10 production inhibits inflammasome activation, impairing inflammasome formation and cell death by pyroptosis and the efferocytosis of infected cells. This phenomenon, in our model, seems to impair an efficient resolution of infection and allow bacterial dissemination

The collective findings demonstrate the *K. pneumoniae*-mediated induction of protective pyroptosis via inflammasome activation (Fig. 8) and strongly suggest the mechanism of inflammasome signaling inhibition through IL-10 production.

## Discussion

IL-1β secretion occurs upon caspase-1 activation due to canonical activation of the inflammasome or non-canonical activation via caspase-11/NLRP3 activation. Inflammasome activation commonly ends in pyroptosis, a process of programmed inflammatory cell death dependent on caspase-1 and/or caspase-11^36^. This pattern of cell death results in the formation of a plasma membrane pore that causes water influx and cell swelling, in addition to the release of cellular contents as LDH and proinflammatory cytokines^37^. Host cell death is a well-spread response mechanism induced by different pathogens during the infection course^38^. *K. pneumoniae* strains are able to survive and proliferate inside macrophages by avoiding phagosome fusion with lysosomes^39^; in the present study, we investigate the impact of caspase-1/11-mediated cell death induced by clinical strains of *K. pneumoniae* on the restriction of bacterial growth.

In the A28006 *K. pneumoniae*-infected BMDMs, both LDH release and PI incorporation were dependent on caspase-1/11. However, these pores are not large enough to allow bacterial escape, and they trigger a PIT that traps bacteria. The pyroptotic process, however, is not able to kill the bacteria^30^, which might explain the elevated in vitro proliferation after the microbicidal protocol. Nevertheless, Jorgensen et al. demonstrated that this process of pyroptotic pore formation damaged bacteria, which became susceptible to microbicidal factors^30^. These findings might explain the elevated resistance of the A28006 strain to hydrogen peroxide when pyroptosis was blocked.

In the same manner as apoptotic bodies, the PIT releases “find-me” and “eat-me” signals, which allow the second phagocyte to locate and recognize the dying cell, resulting in efferocytosis^40–42^. IL-1β, IL-18, and eicosanoids produced after inflammasome activation are some of the mediators responsible for cell recruitment and further efferocytosis^43^. Our findings are consistent with a previous study in that we showed that IL-1β production is connected with the in vitro efferocytosis and in vivo control of bacterial infection, as well as efferocytosis of pyroptotic cells. We observed that efferocytosis of infected pyroptotic cells results in cell activation and production of proinflammatory mediators, which contributes to killing the damaged bacteria inside the PIT, culminating in bacterial clearance, since treatment with Cyto D, which impairs phagocytosis of pyroptotic cells, resulted in bacterial proliferation. Also, when we inhibited the pyroptosis using knockout animals, we impaired the efferocytosis of these dead cells and the A28006 strain is able to proliferate in vitro and disseminate in vivo. Collectively, these data indicate that, according to our model, efferocytosis of pyroptotic cells might be the major process responsible for bacterial clearance in vivo.

Although pyroptosis was initially described as a pathogenic mechanism used by bacteria to evade the immune system^44^, recent studies have shown that this cell death pathway is related to host defense^30,37,45^. These statements are in agreement with our findings, as we showed that the efferocytosis of pyroptotic infected cells might be the main mechanism to explain the bacterial control observed in vivo.

However, some bacteria such as *C. burnetii*^17^ and *Salmonella* Typhi^46^ are able to escape inflammasome activation as a mechanism of pathogenicity that causes host damage and, sometimes, even death. Expression of a gene called *IcaA* by *C. burnetii* allows these bacteria to suppress caspase-11-mediated inflammasome activation induced by *L. pneumophila*. This effector protein is secreted by the Dot/Icm type IV secretion system and inhibits caspase-11-mediated non-canonical activation of the NLRP3 inflammasome. Similarly, *S*. Typhi efficiently evades NLRC4 inflammasome by repressing flagellin expression during systemic infection.

Notably, we observed an antagonistic pattern of inflammasome activation among the multidrug-resistant KPC-producing *K*. *pneumoniae* strains of A28006 and A54970. When we deeply investigated this phenomenon, we observed the capacity of the A54970 strain to negatively modulate inflammasome activation and consequently the pyroptotic cell death, IL-1β and NO secretion, and increase of IL-10 production. Even though the A54970 strain induces high expression of IL-10, we were not able to distinct M1 and M2 phenotypes under this bacterial infection. However, other studies have shown that NLRP3 expression drives the polarization of M2 macrophages in asthma through upregulation of IL-4^47^ and *Il4* promoter binding and transactivation in conjunction with the transcription factor IRF4^48^. Therefore, the absence of a macrophage phenotype can be due to the negative modulation of inflammasome.

Despite that, Ip et al. demonstrated that IL-10 production starts after 2 h of LPS cell activation and it is able to downregulate mammalian target of rapamycin (mTOR) pathway. In addition to controlling glucose and lipid metabolism, the mTOR pathway induces autophagy and cell proliferation, and its inhibition results in mitophagy^35,49^. IL-10 also acts through signal transducer and activator of transcription factor 3 (STAT3), inducing the transcription of anti-inflammatory genes. Both IL-10- and STAT3-deficient mice present prolonged cell activation^35^. Regarding the inflammasome activation, IL-10 can inhibit caspase-1 cleavage through dysregulation of the NLRP3. In this study, we demonstrated for the first time that *K. pneumoniae* strain is capable of inhibiting inflammasome and pyroptosis activation through IL-10 production and its impact on bacterial clearance by modulating efferocytosis of these pyroptotic cells. In addition, as already reported, we also demonstrated that IL-10 negatively modulated the downstream events by diminishing the production of this IL-1β, probably via induction of mitophagy.

Pyroptotic cell death plays an important in vivo role to control and even abolish pathogen replication^50^, which is consistent with our reports. These findings provide the first evidence that pyroptosis is a protective mechanism against KPC-2-producing *K. pneumoniae*, favoring their clearance by efferocytosis. Additionally, we showed that a clinical strain of *K. pneumoniae*, acting through IL-10, inhibits the caspase-1 and caspase-11 activation and their functions. Inhibition of pore formation enables this pathogenic strain to evade microbicidal features and to damage the host due to its dissemination. Finally, it is important to mention that, in light of the rapid antibiotic resistance developed by bacteria, a study that focuses on the evasion mechanisms, such as the one represented in this study, is worthwhile and necessary to create new strategies and alternative therapies for achieving more successful and effective treatments.

## Materials and methods

### Mice

WT C57BL/6 female mice (6–10-week old) were purchased from Multidisciplinary Center for Biological Research, University of Campinas (CEMIB/UNICAMP). Casp1/11^−/−^ female mice (6–10-week old) were kindly provided by Professor Dr. Dario Simões Zamboni (Center for the Creation of Special Mice, University of São Paulo—CCCE/USP). IL-10^−/−^ female mice (6–10-week old) were purchased from CCCE/USP (originated from JAX stock #002251). All animals were housed in specific-pathogen-free conditions within the animal care facility at the Department of Biological Sciences, School of Pharmaceutical Sciences, São Paulo State University with controlled temperature, humidity, airflow, and dark/light cycle with access to sterilized water and food ad libitum. All animals’ experiments performed were approved by the Institutional Animal Care and Use Committee from School of Pharmaceutical Sciences, São Paulo State University (UNESP).

### Bacterial strains

The KPC-2-producing *K. pneumoniae* A28006 and A54970 were previously characterized. A28006 was collected from a Brazilian hospital of Recife in 2006, and it was the first reported producer of KPC-2 isolated in Brazil. A54970 strain was collected from the Brazilian hospital of São Paulo in 2009. Both strains were responsible for causing bloodstream infections in hospitalized patients. According to multilocus sequencing typing characterization, the A28006 and A54970 strains were classified as belonging to ST11 and ST437, respectively. Both strains were resistant to all antimicrobial agents tested, except polymyxin B. In addition, both strains carry *fimH* and *mrkD* genes, which are responsible for codifying fimbrial adhesins of type 1 and type 3, correspondingly. The genes *kpn*, *ycfM*, and *entB*, responsible for codifying FimH-like adhesin, an outer membrane lipoprotein, and enterobactin, respectively, were also detected in both strains as well as *clpK*, associated with thermotolerance, and *tratT*, related to human serum resistance. By contrast, only the A28006 strain possesses the *ybtS* gene, which encodes yersiniabactin, an iron siderophore. *K. pneumoniae* isolates were streaked at 37°C on MacConkey agar containing 5 μg.mL^−1^ of ceftazidime (CZA) for colony isolation and cultivated in tryptic soy broth (TSB) at 37°C under rotation (180 rpm). *E. coli*–ATCC 25922 was streaked at 37°C on Luria-Bertani agar plates without antibiotics for colony isolation and cultivated in Luria-Bertani broth at 37°C under rotation (180 rpm).

### Mice infection and in vivo evaluation of strains' dissemination capacity

WT, Casp1/11^−/−^, and IL-10^−/−^ mice were instilled with 1 × 10^7^ CFU applied to the nose tip of mice and was involuntarily inhaled as previously described by Ye et al.^51^. After 48 h of inoculation, mice were euthanized with isoflurane and the lung and spleen were removed aseptically and homogenized with a tissue homogenizer (IKA - T10 basic ULTRA-TURRAX) in the presence of sterile phosphate buffer saline (PBS) with protease inhibitor cocktail (Sigma-Aldrich). The homogenates were diluted and CFU was recovered in MacConkey agar containing 5 μg.mL^−1^ of CZA. Supernatants from lung homogenates were stored in −80 °C.

### Macrophage culture and infection

BMDMs isolated from WT, Casp1/11^−/−^, and IL-10^−/−^ mice were grown in complete Dulbecco's Modified Eagle Medium (DMEM—Lonza) (10% fetal bovine serum (Gibco), 1% non-essential amino acids (Sigma-Aldrich) and 1% pyruvate (Sigma-Aldrich)) with 20 ng.mL^−1^ of M-CSF (PeproTech) at 37°C, in a humidified incubator with 5% CO_2_, for 7 days. BMDMs were replated into 24- or 96-well plates in complete DMEM medium at a density of 1 × 10^6^ cells.mL^−1^ and infected with the different strains at a multiplicity of infection (MOI) of 5 for 90 min. Cells were washed twice with a cocktail of antibiotics (CZA 5 μg.mL^−1^, gentamicin 5 µg.mL^−1^, streptomycin 12 µg.mL^−1^ and penicillin 12 µg.mL^−1^) and then washed with PBS alone to remove non-phagocytosed bacteria.

### Phagocytosis and microbicidal assay

To assess phagocytosis by CFU, cells were lysed with saponin 0.5% (AcrosOrganics) and CFU was recovered in MacConkey agar (CZA 5 μg.mL^−1^). Phagocytosis was also assessed by flow cytometry following infection with labeled bacteria (5 μM of carboxyfluorescein succinimidyl ester (CFSE) — Thermo Fisher Scientific) for 90 min and incubated with Trypan Blue (1 mg.mL^−1^ in PBS) to quench extracellular fluorescence from non-phagocytosed bacteria. After being quenched, the cells were washed with PBS, harvested with a cell scraper, and fixed with ρ-formaldehyde 4%. CFSE^+^ cells were detected by flow cytometry (BD FACSVerse). For microbicidal assay, cells were reincubated for 4 h before cell lysis with saponin 0.5% and CFU recovery in MacConkey agar (CZA 5 μg.mL^−1^).

### Cytotoxicity assay—LDH release and PI incorporation assay

After 90 min of infection, cells were reincubated for 4 h, and pore formation was determined by measuring LDH release in the supernatant according to the manufacturer's instructions (Promega). Cell pore formation was also confirmed by PI (BD Biosciences) incorporation and analysis by flow cytometry.

### In vitro H_2_O_2_ susceptibility assay

After 4 h of incubation, cells were lysed with saponin 0.5%, and the cell lysates were added to the TSB media and incubated for 2 h in the presence or absence of 0.2, 2, or 20 mM H_2_O_2_, as previously described by Jorgensen et al.^30^. Then CFU were recovered in MacConkey agar (CZA 5 μg.mL^−1^).

### Efferocytosis assay

Cells were labeled with 5 μM of CFSE solution according to the manufacturer’s protocol, and after 90 min of infection, these were reincubated for 4 h. Then new BMDMs were incubated in the presence or absence of Cyto D (10 μM, Calbiochem) and co-cultured infected cells in a ratio of 1:3 (new BMDMs:infected cells) for 2 h. Non-efferocytosed cells were quenched with Trypan Blue, and after quenching, cells were washed with PBS and analyzed by flow cytometry. Bacterial clearance through efferocytosis was assessed by CFU.

### Co-infection assay

Cells were treated with LPS 100 ng mL^−1^ (Sigma) or infected with strains of *K. pneumoniae* during 90 min, then submitted to wash step as described before and reincubated for next 24 h. Subsequently, the cells were infected with *E. coli* (MOI 20) for 4 h, and the supernatant was stored at −80 °C for cytokine detection.

### Pretreatment with IL-10

WT and IL-10^−/−^ cells were treated with recombinant IL-10 (PeproTech) 50 ng.mL^−1^ for 24 h and infected with strains of *K. pneumoniae* during 90 min, submitted to wash step as described before, and reincubated for the next 18 h, and the supernatant was stored at −80 °C for cytokine detection.

### Quantitative real-time PCR (qPCR)

RNA from infected BMDMs was isolated using RNAspin Mini according to the manufacturer’s instructions (GE Healthcare) and reverse-transcribed into cDNA using the iScript cDNA Synthesis Kit (BioRad). Gene expression was determined by amplification with specific primers and quantification by SybrGreen (Life). The relative gene expression was calculated by the 2^−ΔCt^ method. For qPCR, a thermocycler ABI Prim 7500 (Applied Biosystems, Foster City, CA) was used. Primer sequences used to determine macrophage phenotype were: *Arg1* (forward, 5′-CTCCAAGCCAAAGTCCTTAGAG-3′; reverse, 5′-AGGAGCTGTCATTAGGGACATC-3′), *Mrc1* (CD206) (forward, 5′-CCATTTATCATTCCCTCAGCAAGC-3′; reverse, 5′-AAATGTCACTGGGGTTCCATCACT-3′), *Fizz-1* (forward, 5′-ACTGCCTGTGCTTACTCGTTGACT-3′; reverse, 5′-AAAGCTGGGTTCTCCACCTCTTCA-3′), *Cd86* (forward, 5′-CARGGGCTTGGCAATCCTTA-3′; reverse, 5′-AAATGGGCACGGCAGATATG-3′), *Nos2* (iNOS) (forward, 5′-CAGCTGGGCTGTACAAACCTT-3′; reverse, 5′-GCTCTGTTGAGGTCTAAAGGCT-3′), *Gapdh* (forward, 5′-AACTTTGGCATTGTGGAAGG-3′; reverse, 5′-ACACATTGGGGGTAGGAACA-3′), and *Il-6* (forward, 5′-TTCCATCCAGTTGCCTTCTTG-3′; reverse, 5′-AGGTCTGTTGGGAGTGGTATC-3′).

### Enzyme-linked immunosorbent assay (ELISA)

ELISA assay was performed to quantify IL-1β, IL-10, IL-12, and TNF-α (BD Biosciences) from lung homogenate and culture supernatants. The minimum detectable concentrations are 62.5 pg.mL^−1^ for IL-12 (BD Pharmingen) and 31.25 pg.mL^−1^ for IL-1β and IL-10, respectively (BD Pharmingen), and 15.65 pg.mL^−1^ for TNF-α (BD Pharmingen). All procedures were performed according to the manufacturer’s instructions.

### Nitrite and H_2_O_2_ detection

Both mediators were detected in the culture supernatant after 18 h of incubation that was followed by 4 h of incubation. NO was measured indirectly by quantifying nitrite metabolites, as previously described by Griess^52^. H_2_O_2_ was detected as previously described by Muijsers et al.^53^. Briefly, supernatant was incubated in the presence of 25 µM 123-dihydrorhodamine for 15 min in the dark. After incubation, fluorescence was measured by fluorometric analysis as excitation/emission wavelengths of 485 and 530 nm, respectively (BioTek – Synergy H1).

### Statistical analysis

The data were plotted and analyzed using the GraphPad Prism 6.0 software. The statistical significance was calculated using analyses of variance followed by Tukey post hoc tests. Differences were considered statistically significant when *P* value was <0.05.

## Electronic supplementary material

Supporting Information
