# Supplementary material for: Inhibition of inflammasome activation by a clinical strain of Klebsiella pneumoniae impairs efferocytosis and leads to bacterial dissemination
Source: Cell Death Dis. 2018 Dec 5;9(12):1182. doi: 10.1038/s41419-018-1214-5 (PMC6281591; doi:10.1038/s41419-018-1214-5)
Supplement: Supplementary file 1 — Supporting Information [file 41419_2018_1214_MOESM1_ESM.pdf]

SUPPORTING INFORMATION

Supplemental figures

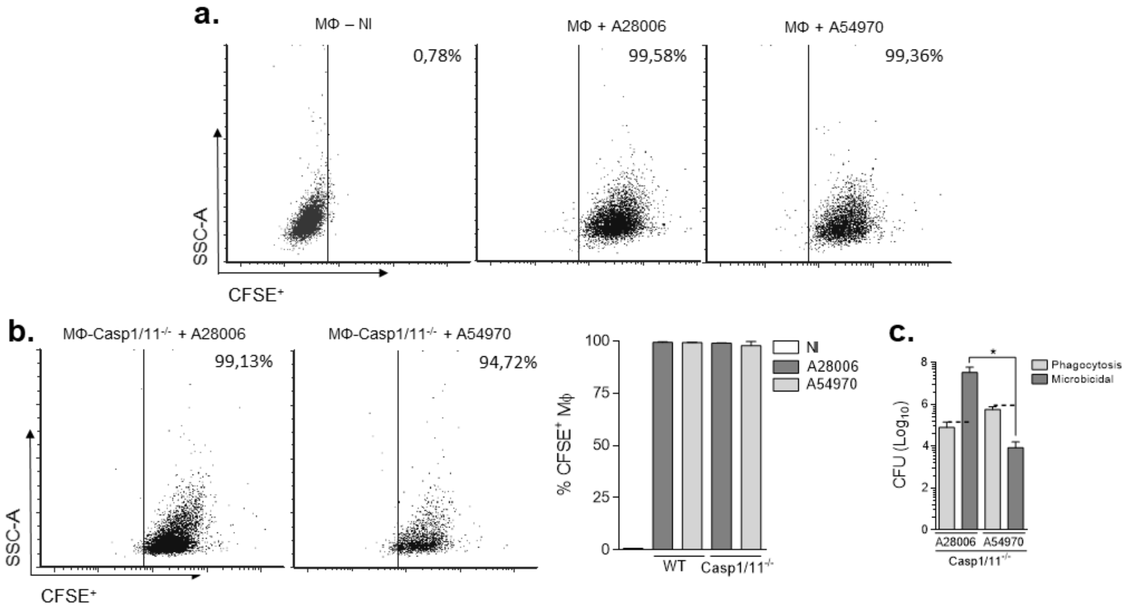

Supplemental Figure 1 | There is no difference in the phagocytic capacity of macrophages against the different KPC-2 producing *K. pneumoniae* strains. a WT or **b** Casp1$^{-/-}$/Casp11$^{-/-}$ BMDMs were differentiated from C57BL/6 mice and incubated with CFSE-labeled *K. pneumoniae* (MOI 1:5) for 90 min. Trypan Blue was added for quenching and the percentage or mean of macrophages CFSE^{+} cells were analyzed by flow cytometry. Dot plot graphic represents the percentage of CFSE^{+} macrophages from three independent experiments in triplicates. **c** Casp1$^{-/-}$/Casp11$^{-/-}$ BMDMs were incubated with the A28006 or A54970 strain (MOI 1:5) for 90 min. Cells were washed twice with a cocktail of antibiotics and one last wash with PBS. For the phagocytosis assay, cells were lysed and CFU was recovered. For the microbicidal assay, cells were incubated for more 4 h before cell lysis and CFU recovery. Statistical analysis: one-way ANOVA and Tukey post hoc tests *$P<0.05$.

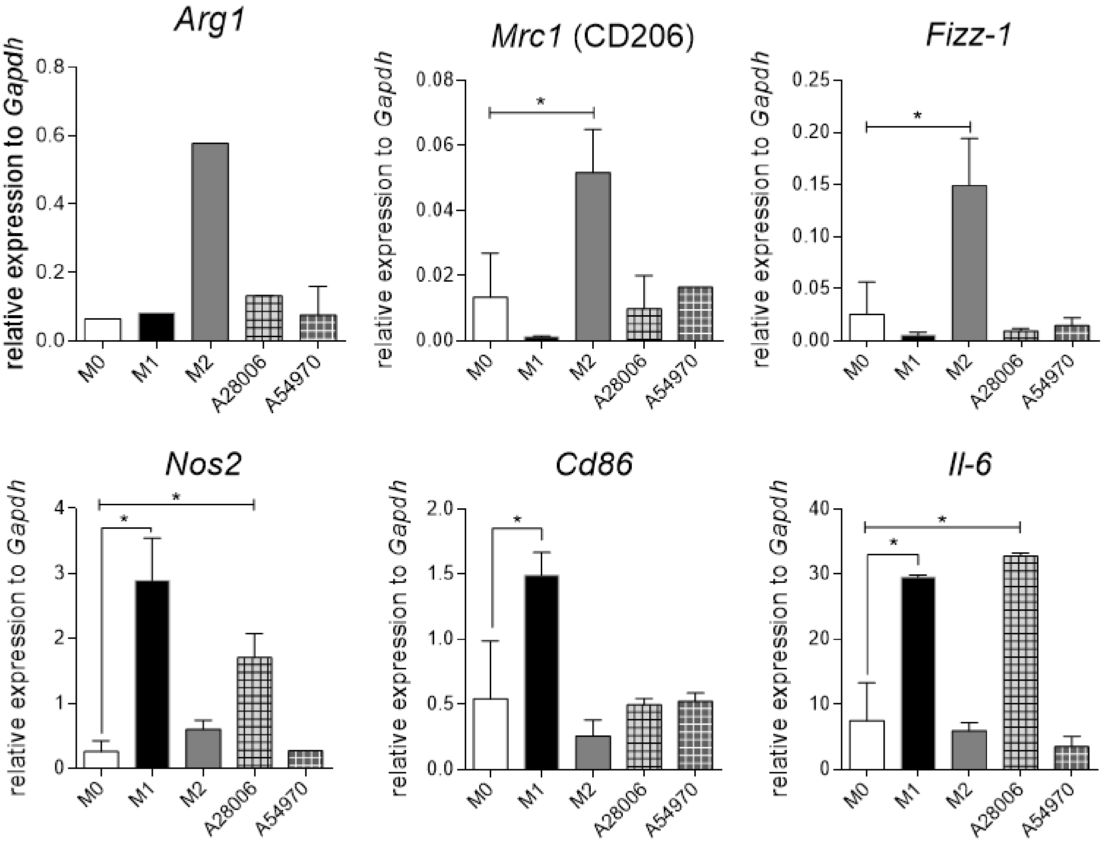

Supplemental Figure 2 | A28006 strain induces M1 macrophage polarization. WT BMDMs were incubated with the A28006 or A54970 strain (MOI 1:5) for 90 min. Cells were washed twice with a cocktail of antibiotics, one last wash with PBS and incubated for more 4 h. Then, cells were collected and mRNA expression was evaluated by qPCR. Data represent the mean ± SEM of two independent experiment performed in triplicate. Statistical analysis: one-way ANOVA and Tukey post hoc tests. *$P<0.05$.

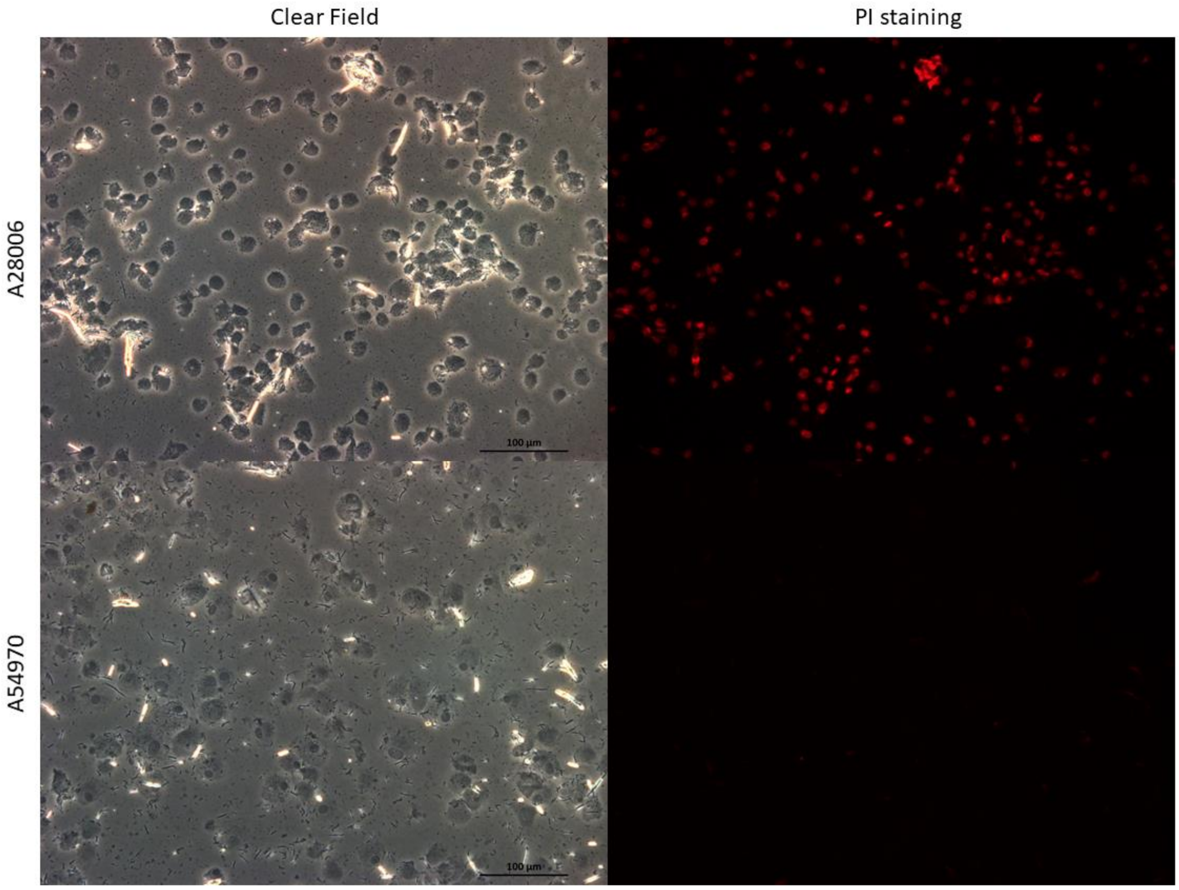

Supplemental Figure 3 | WT BMDMs were incubated with the A28006 or A54970 strain (MOI 1:5) for 90 min. Cells were washed twice with a cocktail of antibiotics, one last wash with PBS and incubated for more 4 h. Representative image of macrophages in clear field and PI staining. Microscopy labeling PI in microscopy magnification of 200x**.**

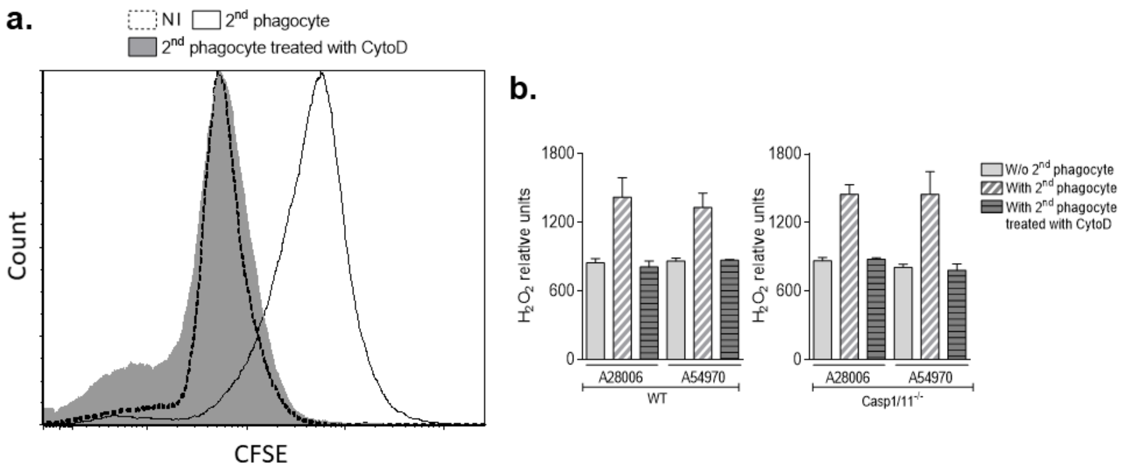

Supplemental Figure 4 | Cytochalasin D inhibits efferocytosis and impairs resolution of infection. WT or Casp1/11$^{-/-}$ BMDMs were incubated with the A28006 or A54970 strain (MOI 1:5) for 90 min. Cells were washed twice with a cocktail of antibiotics and, one last wash with PBS and incubated for more 4 h. **a** BMDMs were labeled with CFSE 5 µM prior to cell infection. After 4 h incubation, new unlabeled BMDMs were treated with cytochalasin D (10 µM) and then co-cultured with infected cells for 2 h in a ratio of 1:3 (new BMDMs:infected cells). Non-phagocyted cells were quenched and washed with PBS. The percentage of CFSE^{+} macrophages was determined by flow cytometry. Inhibition of phagocytosis by cytochalasin D was shown by representative histogram of three independent experiments. **b** After 4 h incubation, new BMDMs were incubated in the presence or absence of cytochalasin D 10 µM and then co-cultured with infected cells for 18 h in a ratio 1:3 (new BMDMs:infected cells). Supernatant was collected and H_2O_2 was quantified by incubation in the presence of 25 µM 123-dihydrorhodamine. Data represent the mean ± SEM of one independent experiment performed in triplicate.

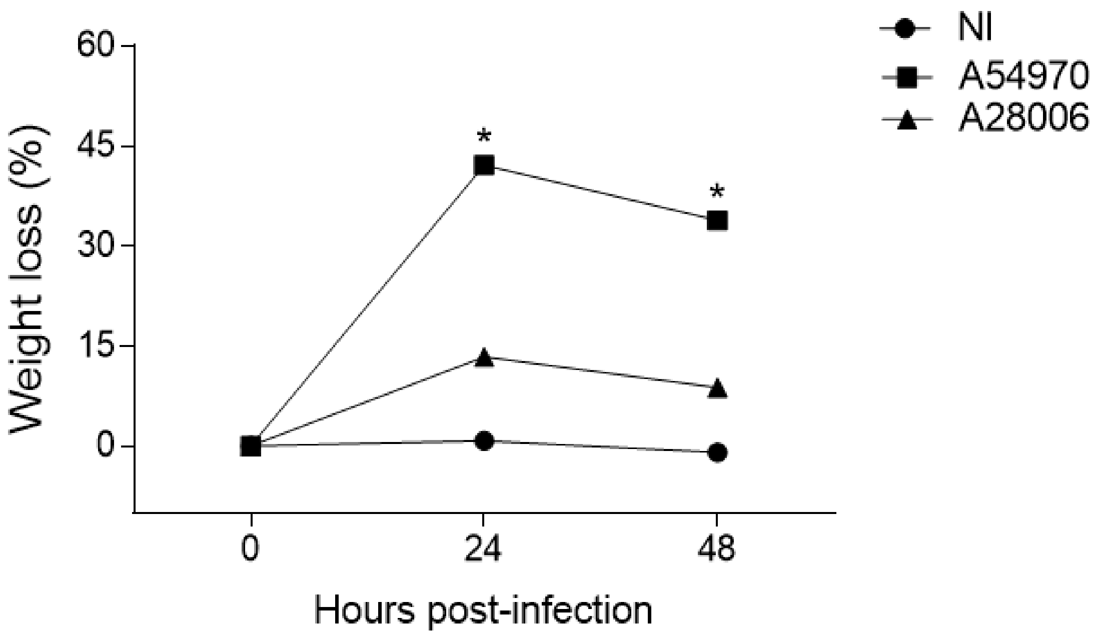

Supplemental Figure 5 | A54970 infection induces weight loss in animals. C57BL/6 mice were nasally instilled with 1×10^7 CFU. Animals were weighed before and 24 and 48 h after instillation. Data represent the mean of one experiment (n=6 animals/group). Statistical analysis: one-way ANOVA and Tukey post hoc tests. *$P<0.05$.
